# Supplementary material for: Social learning through associative processes: a computational theory
Source: R Soc Open Sci. 2019 Mar 13;6(3):181777. doi: 10.1098/rsos.181777 (PMC6458397; doi:10.1098/rsos.181777)
Supplement: Supplementary materials for “Social learning through associative processes: A computational theory” [file rsos181777supp1.pdf]

Supplementary materials for “Social learning through associative processes: A computational theory”

Johan Lind, Stefano Ghirlanda & Magnus Enquist

October 18, 2018

Contents

1 Introduction **1**

2 Learning simulator **1**

3 Simulations **2**

3.1 Learning to respond to social stimuli 2

3.1.1 Learning to respond to a social stimulus 2

3.1.2 Learning to imitate 3

3.2 Learning to respond to non-social stimuli 4

3.2.1 Learning to respond to non-social stimuli 4

3.2.2 Benefits of imitation 5

3.3 Learning behavior sequences 6

3.4 Avoidance learning 8

1 Introduction

This document presents the program and contains the code used for simulations in our paper “Social learning through associative processes: A computational theory”. Together with the learning simulator program, these scripts produce figures[1].

2 Learning simulator

The scripts are to be used in a computer program that has been made to simulate learning phenomena. The simulations are based on defining a world, and

[1] Output data files can also be produced by using export commands. See details in user guide for the learning simulator.

parameters for the organism that is subjected to that world, following the formalism described in our paper. The world is defined according to what stimuli are present and what the consequences are when subjected to these stimuli. The organism is defined according to what behaviors it can perform, what its initial conditions are and parameters that determine memory updates and exploration. Different learning mechanisms can be used. In our paper we used stimulus-response learning in part I and II, and stimulus-response- and stimulus value learning (combining instrumental and Pavlovian conditioning) in part III and IV. The simulator can produce both figures and data. Please note that the figures produced by the scripts are similar, but not identical to the ones in our paper.

The learning simulator can be downloaded at:

`https://github.com/markusrobertjonsson/learning_simulator`.

Running the learning simulator requires Python 3.5 (`https://www.python.org/downloads/`) and Matplotlib (`https://matplotlib.org/`). With Python and Matplotlib installed, the file `lesim.py` opens the learning simulator where the scripts below can be used. For details about the learning simulator and how to run the scripts see the user guide 'ug.pdf' that is included in the learning simulator package.

3 Simulations

All learning parameters used in the simulations are specified in each script. Simulations were done using two different learning mechanisms included in the learning simulator. Simulations were either performed using only updates of stimulus-response associations ('SR' in the scripts) or using both updates of stimulus-response associations and stimulus values ('ga'[2] in the scripts).

The scripts are presented in the order in which they appear in the manuscript. Each script is presented briefly. For rationale and full descriptions of the simulations see text in the paper "Social learning through associative processes: A computational theory". Please note line breaking of long lines in the scripts below. Hard line breaks must be fixed for the scripts to run in the learning simulator.

In some scripts we have included `pass` and `pass value`. These refer to alternative options where the opportunity offered by social stimuli are ignored.

3.1 Learning to respond to social stimuli

Two simulations based on stimulus-response associations and social stimuli.

3.1.1 Learning to respond to a social stimulus

This simulates learning to respond to a social stimulus through updates of stimulus-response associations.

[2] 'ga' is short for genetically guided associative learning.

```
# 1a Learning to respond to a social stimulus (figure 1)

@parameters
{
'subjects'            :1000,
'mechanism'           :'SR',
'behaviors'           : ['0','B'],
'stimulus_elements'   : ['social', 'reward','pass_value','new_trial
    '],
'start_v'             : {('social','0'):2, 'default':0},
'alpha_v'             : 0.1,
'beta'                : 1,
'u'                   : {'reward':25, 'pass_value':2, 'default': 0},
}

@phase {'end': 'social=60'}
NEW_TRIAL    'new_trial'     | SOCIAL
SOCIAL       'social'        | B: REWARD    | PASS
REWARD       'reward'        | NEW_TRIAL
PASS         'pass_value'    | NEW_TRIAL

@run

@figure 'Probability of B'
@pplot ('social','B')     {'steps': 'social'}
@pplot ('social','0')     {'steps': 'social'}
@legend

@figure 'Memory changes'
@vplot ('social','B')    {'steps': 'social'}
@vplot ('social','0')     {'steps': 'social'}
@legend
```

3.1.2 Learning to imitate

This simulates the situation where performing behavior $B1$ (or $B2$, respectively) when observing another individual perform behavior $B1$ (or $B2$, respectively) results in a reward. Through updates of stimulus-response associations behavior $B1$ will be performed when $B1$ is observed and behavior $B2$ will be performed if $B2$ is observed.

```
# 1b Learning to imitate (figure 2)

@parameters
{
'subjects'            : 1000,
'mechanism'           : 'SR',
'behaviors'           : ['0','B1','B2'],
'stimulus_elements'   : ['soc_B1','soc_B2','reward','pass_value','
    new_trial'],
'start_v'             : {('soc_B1','0'):2, ('soc_B2','0'):2,'default
    ':0},
'alpha_v'             : 0.1,
'beta'                : 1,
'u'                   : {'reward':25, 'pass_value':2, 'default': 0},
```

```
}

@phase {'end':'soc_B1=60'}
NEW_TRIAL       'new_trial'         | SOC_B1(.5),SOC_B2(.5)
SOC_B1          'soc_B1'            | 'B1': REWARD  | PASS
SOC_B2          'soc_B2'            | 'B2': REWARD  | PASS
REWARD          'reward'            | NEW_TRIAL
PASS            'pass_value'        | NEW_TRIAL

@run

@figure 'Responses to observation of B1'
@pplot ('soc_B1','0')        {'steps': 'soc_B1'}
@pplot ('soc_B1','B1')        {'steps': 'soc_B1'}
@pplot ('soc_B1','B2')        {'steps': 'soc_B1'}
@legend

@figure 'Memory changes'
@vplot ('soc_B1','0')         {'steps': 'soc_B1'}
@vplot ('soc_B1','B1')        {'steps': 'soc_B1'}
@vplot ('soc_B1','B2')        {'steps': 'soc_B1'}
@legend
```

3.2 Learning to respond to non-social stimuli

3.2.1 Learning to respond to non-social stimuli

Here, learning to respond to a non-social stimulus is simulated. We introduce a compound stimulus, consisting of both a social and a non-social part. Initial values are set so that responding to the social part of the stimulus, for example to approach the social stimulus like a conspecific, results in a reward. This way the social stimulus provides an opportunity to learn about the non-social stimulus which will result in high stimulus-response association values to respond to the non-social stimulus.

```
# Learning to respond to a non-social stimulus (figure 3)
@parameters
{
'subjects'           : 1000,
'mechanism'          : 'SR',
'behaviors'          : ['0','B'],
'stimulus_elements'  : ['x','social', 'reward','pass_value','
    new_trial'],
'start_v'            : {('x','0'):4, ('social','B'):10,'default':0},
'alpha_v'            : 0.1,
'beta'               : 1,
'u'                  : {'reward':25, 'pass_value':4, 'social':10, '
    default': 0},
}

@phase {'label':'social learning', 'end': 'new_trial=100'}
NEW_TRIAL        'new_trial'            | X(0.8),X_SOC(0.2)
X                'x'                    | 'B': REWARD        |
    PASS
```

```
X_SOC             ('x','social')             | 'B': REWARD_SOC      |
    PASS
REWARD            'reward'                   | NEW_TRIAL
REWARD_SOC        ('reward','social')        | NEW_TRIAL
PASS              'pass_value'               | NEW_TRIAL

@phase {'label':'individual learning', 'end': 'new_trial=100'}
NEW_TRIAL         'new_trial'          | X
X                 'x'                  | 'B': REWARD        | PASS
REWARD            'reward'             | NEW_TRIAL
PASS              'pass_value'         | NEW_TRIAL

@run  {'phases':'social learning', 'label':'social learning'}
@run  {'phases':'individual learning', 'label':'individual learning
    '}

@figure 'Probability of responding with B towards X'
@pplot ('x','B')  {'runlabel':'social learning', 'steps': '
    new_trial'}
@pplot ('x','B')  {'runlabel':'individual learning', 'steps': '
    new_trial'}
@legend ('Social learning','Individual learning')

@figure 'Memory changes'
@vplot ('x','B')       {'runlabel':'social learning', 'steps': '
    new_trial'}
@vplot ('social','B')  {'runlabel':'social learning', 'steps': '
    new_trial'}
@vplot ('x','B')       {'runlabel':'individual learning', 'steps':
    'new_trial'}
@legend ('Social learning v(x,B)','Social learning v(social,B)','
    Individual learning v(x,B)')
```

3.2.2 Benefits of imitation

This simulation illustrates how information can be transmitted socially through imitation, as described above.

```
# Transmission of behavior though imitation (figure 4)
@parameters
{
'subjects'              : 1000,
'mechanism'             : 'SR',
'behaviors'             : ['B1','B2','B3','B4','B5','B6','B7','B8
    ','B9','B10'],
'stimulus_elements'     : ['social_B1','x','reward','pass_value','
    new_trial'],
'start_v'               : {('social_B1','B1'):5,'default':0},
'alpha_v'               : 0.1,
'beta'                  : 1,
'u'                     : {'reward':25, 'pass_value':4, 'default':
    0}
}

@phase {'label':'social learning', 'end': 'new_trial=25'}
NEW_TRIAL         'new_trial'                | X(0.8),X_SOC(0.2)
```

```
X                'x'                        | 'B1': REWARD      | PASS
X_SOC            ('x','social_B1')          | 'B1': REWARD      | PASS
REWARD           'reward'                   | NEW_TRIAL
PASS             'pass_value'               | NEW_TRIAL
@run  {'phases':'social learning','label':'imitation'}
@parameters {'start_v': {'default':0}}
@run  {'phases':'social learning','label':'without imitation'}

@figure 'Probability of B1 towards X'
@pplot ('x','B1')    {'runlabel':'imitation', 'steps': 'new_trial'}
@pplot ('x','B1')    {'runlabel':'without imitation', 'steps': '
    new_trial'}
@legend ('Imitation','No imitation')

@figure 'Memory changes'
@vplot ('x','B1')    {'runlabel':'imitation', 'steps': 'new_trial'}
@vplot ('x','B1')    {'runlabel':'without imitation', 'steps': '
    new_trial'}
@legend
```

3.3 Learning behavior sequences

This simulation includes the full genetically guided associative learning model ('ga' in the scripts). This allows for conditioned reinforcement so that previously neutral stimuli can acquire conditioned reinforcement value. This example illustrates how a new behavior sequence towards non-social stimuli can be acquired through social learning.

```
# Learning a behavior sequence x->B1->y->B2->reward (figure 5)
@parameters
{
'subjects'                : 1000,
'mechanism'               : 'ga',
'behaviors'               : ['0','B1','B2'],
'response_requirements'   : {'B1':['x'],'B2':['y']},
'stimulus_elements'       : ['x','y', 'social', 'reward', '
    pass_value', 'new_trial'],
'start_v'                 : {('x','0'):4, ('y','0'):4, ('social','B1
    '): 10,'default':0},
'alpha_v'                 : 0.1,
'alpha_w'                 : 0.1,
'beta'                    : 1,
'u'                       : {'reward':25, 'pass_value':4, 'social
    ':10, 'default': 0},
'omit_learning'           : ['new_trial']
}

@phase {'label':'social learning', 'end': 'new_trial=1000'}
NEW_TRIAL        'new_trial'                | X(0.8),X_SOC(0.2)
X                'x'                        | 'B1': Y              |
    PASS
X_SOC            ('x','social')             | 'B1': Y_SOC          |
    PASS
Y                'y'                        | 'B2': REWARD         |
    NEW_TRIAL
```

```
Y_SOC           ('y','social')          | 'B2': REWARD        |
    NEW_TRIAL
REWARD          'reward'                | NEW_TRIAL
REWARD_SOC      ('reward','social')     | NEW_TRIAL
PASS            'pass_value'            | NEW_TRIAL

@phase {'label':'individual learning', 'end': 'new_trial=1000'}
NEW_TRIAL       'new_trial'             | X
X               'x'                     | 'B1': Y             |
    PASS
Y               'y'                     | 'B2': REWARD        |
    NEW_TRIAL
REWARD          'reward'                | NEW_TRIAL
PASS            'pass_value'            | NEW_TRIAL

@run  {'phases':'social learning', 'label':'social learning'}
@run  {'phases':'individual learning', 'label':'individual learning
    '}

@figure 'Frequency of complete sequences'
@nplot 'reward' {'runlabel':'social learning', 'steps': 'new_trial
    '}
@nplot 'reward' {'runlabel':'individual learning', 'steps': '
    new_trial'}
@legend ('Social learning','Individual learning')

@figure 'w-value of Y (conditioned reinforcement)'
@wplot 'y' {'runlabel':'social learning','steps': 'new_trial'}
@wplot 'y' {'runlabel':'individual learning','steps': 'new_trial'}
@legend ('Social learning','Individual learning')

@figure 'Probability of correct response towards X and Y
    respectively'
@pplot ('x','B1')  {'runlabel':'social learning', 'steps': '
    new_trial'}
@pplot ('y','B2')  {'runlabel':'social learning', 'steps': '
    new_trial'}
@pplot ('x','B1')  {'runlabel':'individual learning', 'steps': '
    new_trial'}
@pplot ('y','B2')  {'runlabel':'individual learning', 'steps': '
    new_trial'}
#@pplot (('x','social'),'B1')   {'runlabel':'social learning', '
    steps': 'new_trial'}
@legend

@figure 'Memory changes, social learning'
@vplot ('x','0') {'runlabel':'social learning', 'steps': 'new_trial
    '}
@vplot ('x','B1') {'runlabel':'social learning', 'steps': '
    new_trial'}
@vplot ('y','0') {'runlabel':'social learning', 'steps': 'new_trial
    '}
@vplot ('y','B2') {'runlabel':'social learning', 'steps': '
    new_trial'}
@legend

@figure 'Memory changes, individual learning'
```

```
@vplot ('x','0') {'runlabel':'individual learning', 'steps': '
    new_trial'}
@vplot ('y','B1') {'runlabel':'individual learning', 'steps': '
    new_trial'}
@vplot ('y','0') {'runlabel':'individual learning', 'steps': '
    new_trial'}
@vplot ('y','B2') {'runlabel':'individual learning', 'steps': '
    new_trial'}
@legend
```

3.4 Avoidance learning

This final simulation illustrates how genetically predisposed responses towards warning signals can result in avoidance learning of a potential predator.

```
# Avoidance learning (figure 6)
@parameters
{
'subjects'                : 2000,
'mechanism'               : 'ga',
'behaviors'               : ['escape','0'],  # 0 = ignore
'stimulus_elements'       : ['predator','warning','injured','
    new_trial', 'end'],
'response_requirements'   : {'escape':['predator','warning']},
'start_v'                 : {('predator','escape'):-2,'default':0},
'alpha_v'                 : 0.1,
'alpha_w'                 : 0.1,
'beta'                    : 1,
'behavior_cost'           : {'escape':1, 'default':0 },
'u'                       : {'warning':-50, 'injured':-500, 'default
    ':0},
'omit_learning'           : ['new_trial']
}

@phase {'label':'social_learning', 'end': 'new_trial=100'}
NEW_TRIAL         'new_trial'                 |SELF(0.5),SOCIAL(0.25),
    SOCIAL_WARNING(0.25)
SELF              'predator'                  |'escape': END  |END(0.2)
       |ATTACK(0.01)  |SELF
SOCIAL            'predator'                  |'escape': END  |END(0.2)
       |ATTACK(0.01)  |SOCIAL(0.5),SOCIAL_WARNING(0.5)
SOCIAL_WARNING    ('warning','predator')      |'escape': END  |END(0.2)
       |ATTACK(0.01)  |SOCIAL(0.5),SOCIAL_WARNING(0.5)
ATTACK            'injured'                   |END
END               'end'                       |NEW_TRIAL

@phase {'label':'individual_learning', 'end': 'new_trial=100'}
NEW_TRIAL    'new_trial'    |SELF
SELF         'predator'     |'escape': END  |END(0.2)  |ATTACK(0.01)
       |SELF
ATTACK       'injured'      |END
END          'end'          |NEW_TRIAL

@run  {'phases':'social_learning', 'label':'social'}
@run  {'phases':'individual_learning', 'label':'individual'}
```

```
@figure 'Probability of escaping from the predator'
@pplot ('predator','escape')    {'runlabel':'social', 'steps': '
    new_trial'}
@pplot ('predator','escape')    {'runlabel':'individual', 'steps':
    'new_trial'}
@legend ('Social learning','Individual learning')

@figure 'Memory changes, social learning'
@vplot ('predator','escape')   {'runlabel':'social', 'steps': '
    new_trial'}
@vplot ('predator','0')        {'runlabel':'social', 'steps': '
    new_trial'}
@legend ('Predator -> Escape','Predator -> Ignore')

@figure 'Development of w-values, social learning'
@wplot 'predator'    {'runlabel':'social','steps': 'new_trial'}
@wplot 'warning'  {'runlabel':'social','steps': 'new_trial'}
@legend

@figure 'Frequency of being injured (per encounter)'
@nplot 'injured'  {'runlabel':'social', 'steps': 'new_trial'}
@nplot 'injured'  {'runlabel':'individual', 'steps': 'new_trial'}
@legend ('Social learning','Control')
```
